# Co-Authorship and Bibliographic Coupling Network Effects on Citations

**DOI:** 10.1371/journal.pone.0099502

**Published:** 2014-06-09

**Authors:** Claudio Biscaro, Carlo Giupponi

**Affiliations:** 1 Department of Economics, Ca' Foscari University of Venice, Venice, Italy; 2 Department of Management, Ca' Foscari University of Venice, Venice, Italy; 3 Institut für Organization und Globale Managementstudien, Johannes Kepler Universität, Linz, Austria; The Centre for Research and Technology, Hellas, Greece

## Abstract

This paper analyzes the effects of the co-authorship and bibliographic coupling networks on the citations received by scientific articles. It expands prior research that limited its focus on the position of co-authors and incorporates the effects of the use of knowledge sources within articles: references. By creating a network on the basis of shared references, we propose a way to understand whether an article bridges among extant strands of literature and infer the size of its research community and its embeddedness. Thus, we map onto the article – our unit of analysis – the metrics of authors' position in the co-authorship network and of the use of knowledge on which the scientific article is grounded. Specifically, we adopt centrality measures – *degree*, *betweenneess*, and *closeness centrality* – in the co-authorship network and *degree*, *betweenness centrality* and *clustering coefficient* in the bibliographic coupling and show their influence on the citations received in first two years after the year of publication. Findings show that authors' *degree* positively impacts citations. Also *closeness centrality* has a positive effect manifested only when the giant component is relevant. Author's *betweenness centrality* has instead a negative effect that persists until the giant component - largest component of the network in which all nodes can be linked by a path - is relevant. Moreover, articles that draw on fragmented strands of literature tend to be cited more, whereas the size of the scientific research community and the embeddedness of the article in a cohesive cluster of literature have no effect.

## Introduction

Generating scientific knowledge is as a social activity in which scientists find problems to tackle, become aware of connections between elements, elaborate on existing ideas to produce new or refined answers. It is a recipe with both social and knowledge ingredients. However, studies on the impact of scientific knowledge have mostly stressed either the social or the knowledge part of the story, neglecting their interaction.

Studies on co-authorship networks have gathered the attention of many scholars, not just because of their descriptive and synthetic power to describe the evolution of research communities, but also because social networks play a significant role on the generation of knowledge [1]–[3]. Social interactions still play a major role even in an era in which knowledge is accessible on-line [4], [5], in contrast of what common sense would suggest. Co-authorship networks (hereafter CA) are a type of social network based on co-author relationships that are built over time by scientists.

The increasing number of researches that link CA and scientific impact – citations – reminds us that collaboration and its structure profoundly affect the quality of work. In this respect, scholarly attention increased towards the properties of CA and their effects on citations [2], [6], [7]. Newman explored the advantage of the first publications in a field [8], Mazloumian and colleagues documented the bandwagon effect of accolades, world-class recognitions, and landmark papers on prior works of the rewarded scientists [9]. With respect to the network position of co-authors, centrality measures significantly correlate with the article citation count [7]. In particular, degree centrality – that counts the number of personal contacts of an author – and betweenness centrality [2] – that measures how many shortest paths connecting any two authors in the dataset run through any single node.

Co-authorship relations represent the social side of the generative activity of scientists. For individuals, whose main capital and product is knowledge, social interactions are a crucial to improve the quality of their work. They are so relevant that opportunities of unintentional interaction are favored (or even forced) in some contexts oriented to knowledge generation, such as high-tech firms [10], [11]. The reason is that the quality of ideas and knowledge work is given not just by the number of people who create it, but mainly by the knowledge diversity to which they are exposed. Establishing social connections with diverse groups enables the exposure to multiple and different intellectual domains, methods, perspectives and techniques [12], [13] and the inclusion of “whole domains of elements … into the combinative hopper” (Simonton, 1995: 473). Recent works showed that also in different fields of science the attitude towards interdisciplinary is rewarded with more citations [2], [7], [15].

We need a different lens to overcome the paradox in which studies on knowledge generation are mainly carried out by studying the social component, neglecting the most fundamental one: knowledge itself. After all, even the social part of the theory assumes that it is by combining distant or multiple domains of knowledge that new and impactful ideas are generated. Yet, knowledge remains just an outcome. We think that the bibliographic coupling network is the appropriate lens to look at how knowledge is combined into a scientific work. The bibliographic coupling network (hereafter BC) is constructed on the shared references among publications [16], thus it provides deeper insights on the scientific activity, as it reveals information on how authors use and construct links among the existing literature. It can be used to analyze the articles' position within the literature, to infer the size of its research community and it may help answer conjectures that have not yet been addressed.

Indeed, the idea of analyzing the knowledge used by authors is not revolutionary, and it could be traced back at least to the intuition of John of Salisbury, then quoted by Newton who wrote that he was able to see further as he stood on giants' shoulders. This inspiring portrait of a scientist who builds on other scientists' work reveals that scientific work is in an ongoing evolution and there exist a real (and a spiritual) connection among scientists. Along the thread of scientific evolution, Polanyi [17] noticed that scientists adjust their efforts on the basis of “the hitherto achieved results of the others” (p. 2) thus creating a continuum of different works that results in a continuous progress that links different scientific domains and whereby the key knowledge sources change over time [18]–[22].

For this reason, we want to incorporate the analysis of the use of knowledge sources – the references – into the more traditional variables that look at the social interaction in order to study the effects of both social and knowledge networks on the scientific impact of knowledge production. Thus, we integrate metrics of BC and CA to see their effect on articles' citations. Thereby, as we follow this intent, we align with the recent work of Uddin, Hossain and Rasmussen [2], as our unit of analysis is the publication whereby we map metrics from the two network.

To better capture network effects, we isolate papers of a specific literature of vulnerability in climatic change and exclude those outside that literature, whose citations could have a different distribution [23], [24]. We do that by performing an unsupervised textual analysis and categorization of all papers into topics [25]–[27]: this step also allows us to identify elements of originality and innovation in order to control for advantages coming from the introduction of new research topics or new combinations of topics. Then, we construct the CA and BC network [28], to answer the following research questions: (1) How does the co-authorship network structure influence the scientific impact of an article in terms of citations? (2) How do the article's knowledge sources influence its scientific impact?

In this work we use the terms paper, article, publication and work interchangeably. Sometimes the term node or vertex will be referred to the author/co-author or to the paper/article according to the network under scrutiny is the CA or the BC, and, similarly, the term tie or edge will be referred to the co-authorship relation or to the fact that two articles share at least one reference.

The rest of our article proceeds with the description of the methods with which we constructed the dataset and addressed our research questions, than we describe the measures we used and the theoretical reasons underpinning their adoption. Next, we will discuss our methods and research setting. After that we will present the results of our analysis and we will conclude deriving general theoretical contributions.

## Methods

To test our arguments, we need a set of articles that originated within a coherent body of literature. Thus, after downloading the dataset, our first intent is consolidate the set by screening out those publications which entered our search due to the fact that the multiple keyword combinations adopted could in part be used also in contexts far from our interests, like the medical one. We carry out a machine learning classification task [25], [27], [29] on the textual information (more information both on method and on the process is contained in File S1), and isolate only the publications coherent with the identified literature. Only then, we generate two types of networks based on scholarly collaborations, and on articles' references.

### The bibliographic coupling and co-authorship networks

The structure of a scientific literature and that one of collaboration between authors can be analyzed through networks that are based on the mathematical mapping of relations (edges) between dyads of elements (vertices or nodes).

From a set of scientific articles, it is possible to establish relations among different attributes, e.g., references, co-authors, keywords. In this research, we focus on two types of networks: the co-authorship network (CA), and the bibliographic coupling network (BC). In the BC, the articles are the vertices and an edge is established when they have at least one shared reference. Analogously in the co-authorship (CA) network, vertices are the authors and the edges are established between vertices who co-authored an article. We treat the BC as unweighted: the weight of the edge is not affected by the number of shared references.

**Figure 1:**
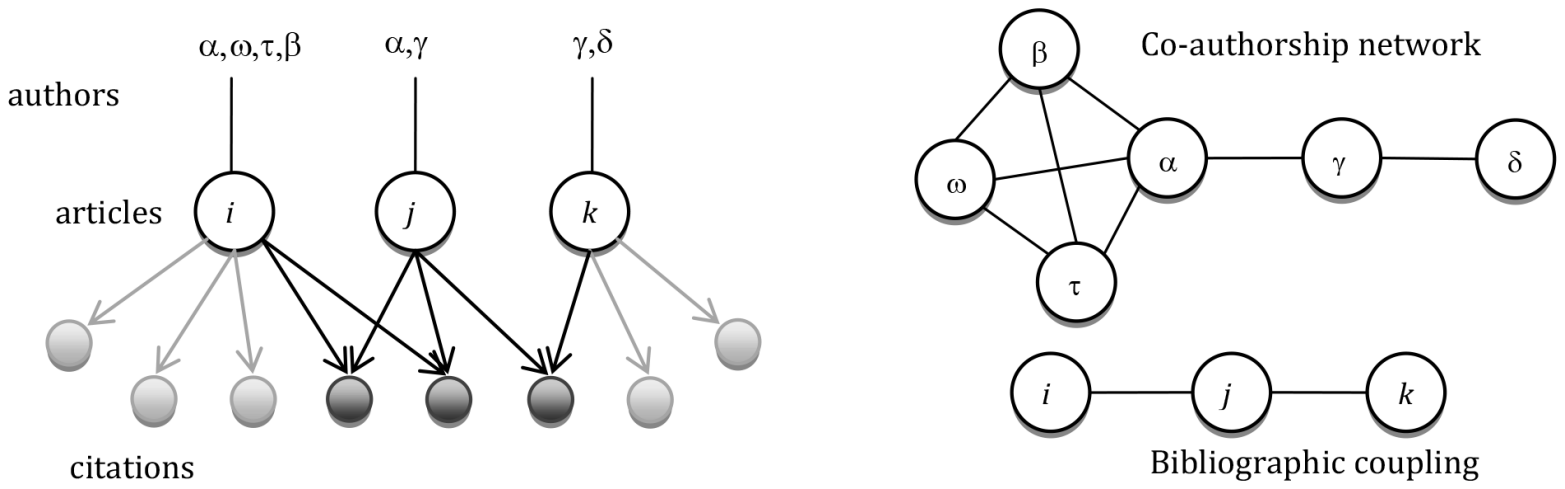
Example of bibliographic coupling network and co-authorship network stemming from a set of three articles.

In Figure 1, we represent how to construct the two networks starting from a set of three articles *i*, *j*, and *k*. With respect to the CA, the activity of co-authoring both in *i* and *j*, enables author α to span across two sets of co-authors β, ω and τ (through article *i*) and γ (through article *j)*.

Generally the choice on networks of citations privileges the choice of visualizing the links among citing and cited articles. This type of network, called co-citation, provides a picture that changes in time and in which there are as many nodes as the number of citing and cited articles. To have a more compact and time-independent network, we choose to establish relations among those articles in the dataset that share references, as shown in Figure 1, where two same references are contained in articles *i*,*j*, while *j* and *k* share one. Thus, the BC contains only three nodes and two edges. Node *j* is linked with and *i*, and with *k* on the basis of the shared references, while there is no edge between *i* and *k*.

The choice of BC has also another key advantage with respect to the co-citation network. Other than compactness of the network (3343 nodes versus over 150,000), the relations that the node (article) establishes with the rest of the network (with the extant literature) is grounded in what the authors decided to include in their reference list. Thereby it is established by means of authors' choices. One of the disadvantages of the BC is that the number of relations depends on the size of the reference list, giving a positive bias towards those articles that have a larger reference list. However, we will control for it as described in the § Measures.

### Data Source

As the scientific impact is dependent on the stream of literature, we build the dataset through a sequence of steps aimed at gathering a homogenous population of articles in terms of thematic structure, information on references and co-authors. The first step consists in the identification of literature streams and it is done through an accurate selection of keywords reported in Table 1. The choice of keywords has dealt with the concept of *vulnerability* that is a crucial notion for several research communities: particularly for climate change adaptation and disaster risk reduction. A positive note on the literature we chose is that the meaning of vulnerability varies in the diverse research streams, which developed in isolation. Only recently, under the push of international bodies (such as IPCC and UNISDR), these fragmented scientific streams are unifying their glossary. Thereby, this is a rich context whereby to test our approach. The focus of scientists spans over a multitude of aspects that vary from strategies to adapt and anticipate possible consequences of a natural extreme event to strategies to better cope with their effects in a multitude of different social or ecological systems, affected by diverse types of natural events (floods, draughts, storms, heat waves, and extreme wind, among the others), and mediated by various morphological, geological, and social conditions.

**Table 1:** Keywords.

Vulnerability & risk assessment
Vulnerability & risk management
Vulnerability & adaptive management
Vulnerability & water resource management
Vulnerability & climate
Vulnerability & climate change
Vulnerability & climate change adaptation
Vulnerability & disaster risk
Vulnerability & disaster risk reduction

The search, performed on April 30 2013, in the ISI Web of Knowledge returned 5585 papers published between 1985 and 2013.

**Figure 2:**
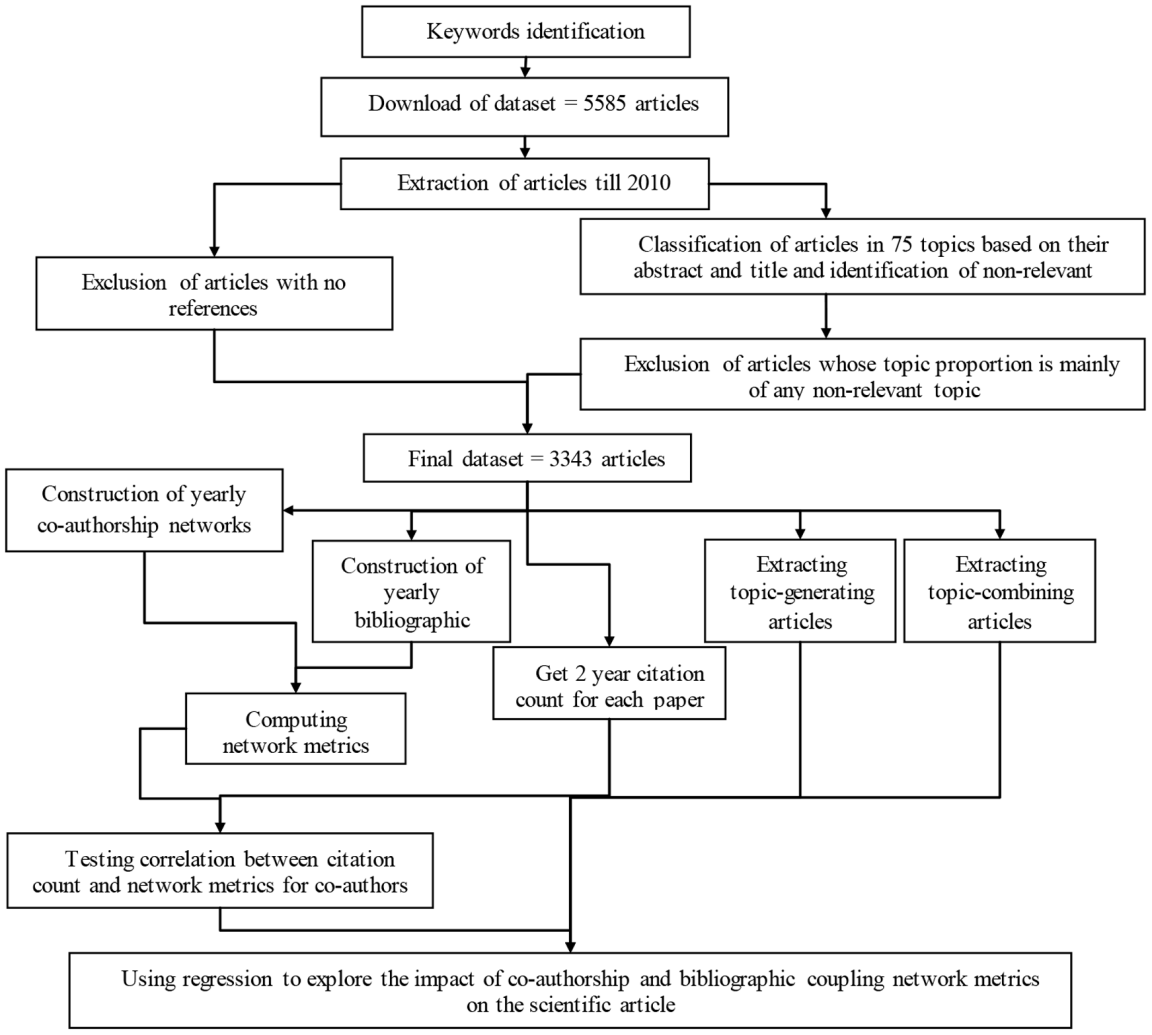
Mapping the process of data analysis.

We refine the dataset in three steps. We select articles that have at least two entire years of forward citations (thus we exclude articles published after 2010), belong to the same scientific ‘macro-discipline’ (the entire process of data analysis is summarized in Figure 2), and have references.

First, we discard the articles published after the 2010, because they do not have two complete years of citation history that is the measure of scientific impact chosen. We choose a 2 year time window as all articles belong to the same disciplinary area and we know that they should have analogous citation patterns.

Second, using the Stanford Topic Modeling suite, we classify articles based on the latent structure of topics extracted from the textual information available from the search: abstracts and titles (we do not consider keywords, as not all journals require them). The algorithm needs to arbitrarily set the number of topics. It is set to 75, a number that takes into account the large variability of the themes discussed in the field: types of environments, receptors, and units of analysis. Topics are coded by the second author of this paper, who is expert in the field (with more than three publications), and labeled on the basis of their 20 most likely words (see the SI for details). Such coding is then validated by two other experts, who also identify nine non-relevant topics. This leads to the exclusion of 270 articles whose content – topic proportion >0.5 – falls in one of these nine topics (see the SI for details).

Third, we exclude articles for which it is not possible to compute the BC network for having no references.

**Figure 3:**
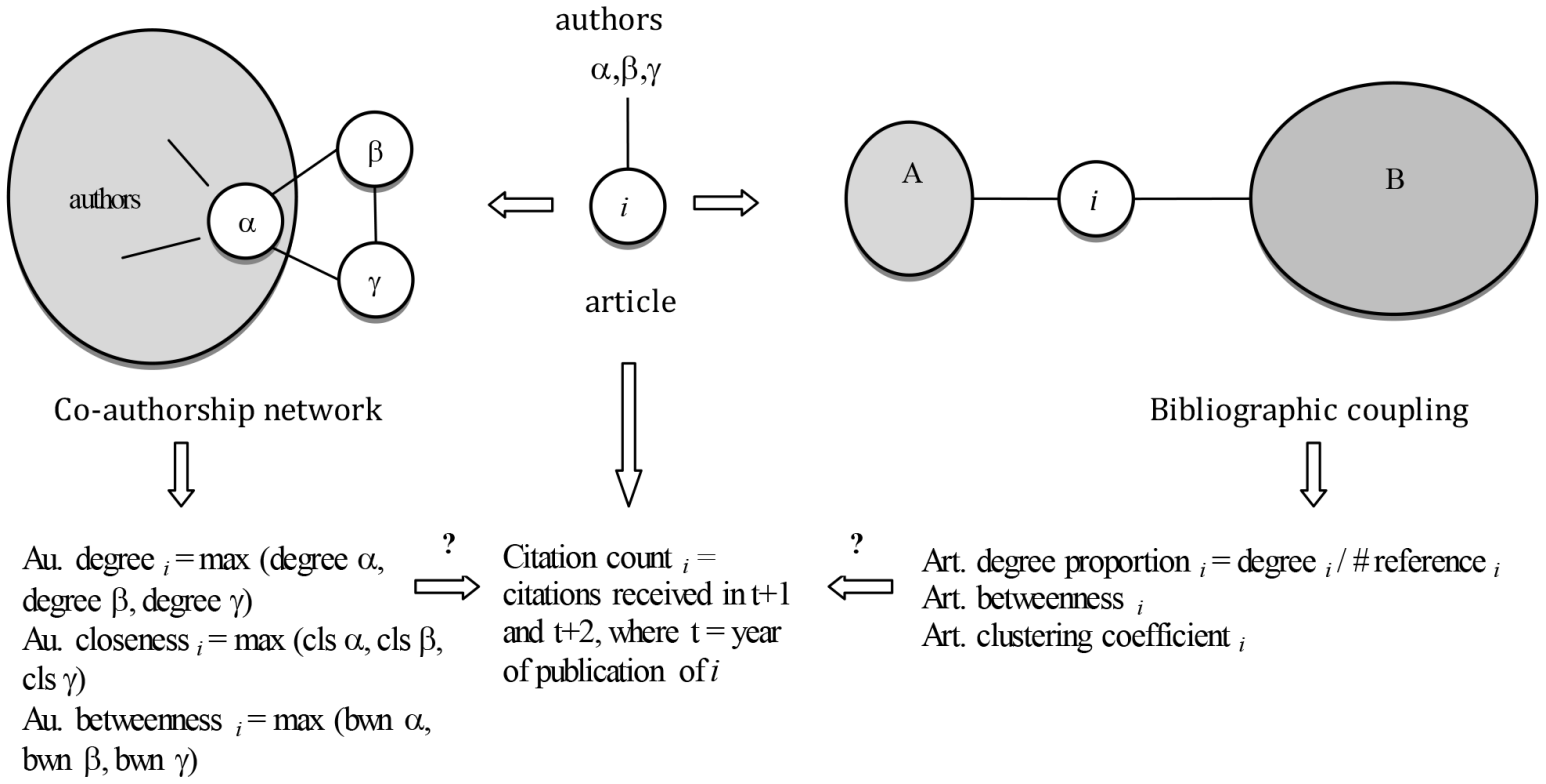
Conceptualization of the research questions. Starting from the article *i* written by α,β and γ and published in year t, two networks are derived. On the left side, the co-authorship network shows that α is already part of a group of other authors and had joint works with two of them (represented by the two edges inside the author set). β and γ are connected to the set of authors through α by means of their new joint-work *i*. We then extract three measures, degree, closeness and betweenness for each co-author of *i*, and map only the maximum value into *i*. On the right side, the article *i* have common references with articles of the sets A and B. We measure the degree, betweenness centralities and clustering coefficient of the article *i*. We construct the dependent variable cumulating the citations received by article *i* in the two years following the year of its publication. Last, we verify the effects the co-authorship and bibliographic coupling networks measures on the citation count of article *i*.

This process brings the dataset to a population of 3343 articles published between 1989 and 2010. We then create a series of BC and CA networks on temporal slices that keep fixed the first observation in the dataset and move forward by one year (e.g., 1989–2002, 1989–2003, 1989–2004).The temporal slices enable us to observe the position of the authors in the CA and the article in the BC at the time of the article publication. For each network slice, we extract our independent variables by means of the library ‘igraph’[30] running in the statistical software R. Last, we performed the statistical analysis by means of negative binomial regressions. In Figure 3, we provide a visual conceptualization of our research questions.

### Measures

#### Scientific impact

The dependent variable is the articles' scientific impact measured by the number of citations received (hereafter *citation count*) within a two-year fixed time window starting after the year of publication. We decide to exclude the citations received during the year of publication, as that would create a bias in favor of the articles published early in the year. A fixed time window to measure citations has been chosen for several reasons: it is shown to be a good measure to determine the scientific impact within a discipline [23]; it is not biased in favor old articles that can be cited for a longer period as it does not fully reflect the ranking of the most cited overall (0.68 spearman correlation coefficient with the cumulative citations received in April 2013); and with respect to the yearly citation average, efficiency [31], it has the advantage to be limited in time, therefore it focus only on the immediate impact of the social activity of co-authors and positioning of the article, while being not affected by phenomena such as drifts in the literature that could impact citations but are also slower to occur. However, the two-year time window is correlated with efficiency (0.84 with p<0.001).

***Degree centrality*** is the count of the first neighbors of a node. In the CA, it equates with the number of co-authors with whom any author has collaborated at the end of year *t* (hereafter *author degree)*. We normalize the count in the BC network, as we use the *degree proportion* that is the degree centrality of article *i* divided by the number articles cited by *i*. Thereby keeping constant the degree of article *i*, its *degree proportion* will decrease with the size of its reference list.

***Closeness centrality*** considers how close the node is to any other node in the network. Therefore a high closeness score demonstrates a short distance between the node – on which it is computed – and any other node. For a single node *i*, it is the inverse of the mean distance of the geodesic (shortest) path *g* to any other.
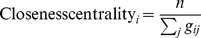



***Betweenness centrality*** of a node *i* is computed summing the number 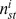 of geodesic paths between any two nodes *s* and *t* that pass through *i* over the total number 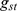 of geodesic path between the two nodes. This is normalized by dividing by the number of ties between any other two nodes.
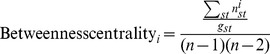



An author *i* with a high betweenness centrality score acts as the shortest path between many other actors, thus potentially benefiting from brokering advantages, especially when the other actors are disconnected if node *i* is removed.

***Clustering coefficient*** of a node *i* is the number of f neighbors of *i* that are connected between each other divided by the number of pairs of neighbors of *i*. The measure, computed only for the BC networks, captures the embeddedness of an article in the existing literature. High values of clustering coefficient show that the articles with which *i* shares references, also share references among them.

Our unit of analysis is the article, therefore we retrieve the only the maximum value for each measure of the CA networks as they represent the value of the most influential co-author who transfers the highest value of authority to the paper [9].

All metrics are computed on yearly slices to capture the values at the year of publication.

### Control variables

#### Topic generation

An article generates a topic when the proportion of text attributed to a topic is for the first time larger than 25%. When in the same year and in the same topic, the threshold is surpassed by multiple articles, all of them are classified as topic-generating. For example, Strzepek et al. [32] and Jose et al. [33] brought in the same year the concept of vulnerability to the context of *water resources and river basins* and we classify both as topic generating. Their proportion of words related to the topic of *water resources and river basins* are beyond the threshold and there is no prior article that accomplishes that. A similar operationalization has been performed by Kaplan and Vakili [34] who considered a less conservative threshold of 20%. Topic generation is a dummy variable that assumes value 1 when an article generates a new topic and 0 otherwise. With this variable, we control for those papers that introduce a certain topic in the field that could be the most important and be rewarded by higher citations [35], almost regardless of their quality [8]. By opening a new research stream, they benefit from a citation advantage over the followers as they lead future research that will necessarily cite them.

#### New topic combination

Similarly to topic generation, articles introduce a new topic combination when they establish a combination of topics that was not present in the dataset until the year of publication. We adopt the same rule used to attribute topic generation: a topic proportion beyond the threshold of 25% determines the presence of that topic in the article and the rule of the multiple attribution of a topic combination applies similarly to *topic generation*: all those articles that introduce the same topic-combination in the same year are identified as introducing a new topic combination. Topic combination controls for the effect of those articles which generate a combinatorial type of innovation [36] at the level of topic.

For robustness, results do not significantly change when checked with thresholds of .20 and .30.

#### Size of the citing literature

Citations depend on the size of the universe of article from which citations are drawn; therefore there is a need to control for the size of this expanding universe. As a proxy of the expanding universe of articles, we take the number of articles in the dataset two years after the year of publication of each article. Although we recognize that this universe must not be exact universe of citing articles, it provides the sense of growing attention towards the topic of vulnerability and, secondly, it is a monotonically growing body of scientific literature, such as the entire universe of scientific literature. For robustness, we also tried two different versions of the metric: (1) we inflated the measure with a relatively large fixed number representing the articles outside the dataset (10,000) that could be interested in referring to articles within; (2) we increased that fixed number (10,000) by a 4.1% each year as it is a plausible rate of growth of scientific articles in the period between 1990–2007 [37]. Results proved to be robust.

#### Number of authors

The number of authors is positively correlated with the *citation count*, see Table 2, and there may be several reasons: a paper with multiple co-authors is more likely to be more complex in terms of knowledge sources, as it required the work of multiple actors; the quality of the content can also be enhanced by the *labor limae* that can be performed by multiple hands; furthermore, the dissemination of the ideas included in the article can spread in the co-authorship network starting from multiple starting nodes. Thus, we sift out the effect by including the number of authors in the subsequent statistical analysis.

**Table 2:** Spearman correlation coefficients with significance level.

	Citations	2	3	4	5	6	7	8	9
(1) Citations									
(2) Author degree	0.34†								
(3) Author betweenness	0.31†	0.81†							
(4) Author closeness	−0.11†	−0.09†	−0.03						
(5) Article degree proportion	0.22†	0.19†	0.28†	0.11†					
(6) Article betweenness	0.30†	0.25†	0.29†	−0.11†	0.70†				
(7) Clustering coefficient	0.15†	0.18†	0.23†	−0.30†	0.56†	0.39†			
(8) Number of authors	0.22†	0.70†	0.38†	−0.07†	−0.02	0.08†	0.03		
(9) Size of Literature	0.18†	0.29†	0.28†	−0.93†	−0.02	0.17†	0.34†	0.15†	
(10) Author's experience	0.28†	0.77†	0.88†	−0.07†	0.32†	0.32†	0.27†	0.25†	0.28†

Levels of significance: † p<.001.All measures are computed at the year of publication of each paper.

#### Experience in the field

Experience is associated with a higher level of specialization, knowledge of the relevant problems in the field and with a deeper ability in publishing and diffusing ideas [38]. Thereby expert authors have both cognitive and reputation advantages. They are better known within the field, have consolidated relationships in the research community, and know how to make networking better than their less-expert peers. This should translate into higher quality production and more effective dissemination. We operationalize the experience in the field for any article *i* by measuring the number of articles in the dataset published by each co-author prior to the publication of *i*. Among these figures, we chose the maximum, as we think that it is the most expert author who has the highest influence on the paper's impact.

#### Citing review bias

In several scientific disciplines there is a high concentration of reviews among the most cited articles [1]. Notwithstanding the difficulty to discriminate between review and non-review papers based solely on the number of references, because to introduce a new conceptual framework to analyze new data, often authors draw on multiple contributions in the extant literature, yet, to control for the citing bias towards articles with a large number of references, we clustered articles in two groups (review, non-review) based on the number of article cited in their reference list. The two groups have profoundly different means (µ_review_ ≈37, (µ_non-review_ ≈106), thus we created a dummy variable with value 1 if the article is a review, 0 otherwise.

#### Bandwagon effect

Citations are boosted by the peer recognition of the author. As already noticed, there is an effect of world-class recognitions and landmark papers on the citations of both prior and subsequent works of the authors [9]. With no availability of data on scientific prizes and accolades for the authors, we decided to control for the effect of peer-recognition by identifying the 309 authors of the articles that were most cited at the end of 2012 (top 1%, 33 articles), and put a dummy variable on the 199 articles written by them in the period starting two years before their landmark paper, with the exclusion of their landmark one. The two year time-window, in which we count citations, makes articles written before that period unaffected by posterior success, as the citing authors should not be probably aware of subsequent success (except for circulating working papers that we cannot control).

To understand the impact of the variables on the *citations count,* we perform negative binomial regressions – a regression model specific for count data in which the dependent variable has overdispersion – with nested sets of regressors. Results are presented in the next section.

## Results

In this section, we first provide an overview of the data, and then we answer the research questions and show how the *citation count* is affected by the structure and positions of co-authors within the co-authorship network, and by the position of the article in the literature.

### Summary statistics

**Figure 4:**
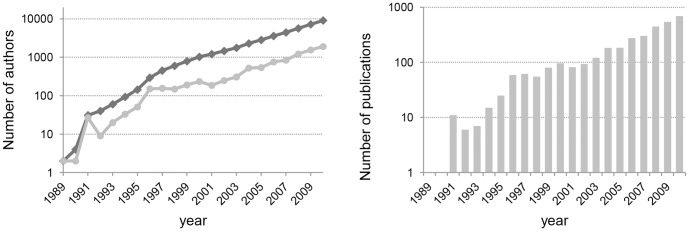
The expansion of the Vulnerability literature: number of authors (left) and publications (right) per year. On the left part of the figure, the dark line represents the cumulative number of authors who at least have one publication in the dataset, while the light line represents the number of new authors.

After a handful of publication in the early 1990s, the literature on vulnerability increases steadily over the years, as displayed in Figure 4 (left). From 2004, the number of papers increases in a steep-log phase. Analogous is the trend of co-authors displayed in Figure 4 (right). In dark grey the cumulative number of authors in the dataset, while in light grey the number of new authors entering in the dataset at any year. Most articles are written by 3 authors (µ = 3.404, σ = 3.021, min  = 1, max  = 57), they have on average 48.65 references (σ = 34.537, min  = 1, max  = 398), and receive on average 4.95 citations in the first two years after the publication (σ = 9.890, min  = 0, max  = 273), however the distribution of citations is skewed to the right.

For the non-normal distribution of most variables in the study, we use Spearman correlation to compute the correlation coefficients between the *citation count* and the independent variables, as shown in Table 2. Correlations show that citation count is positively associated with most independent variables with the expection of *author closeness*. The negative correlation between *Author closeness* is particularly interesting and needs further analysis, because it means that the impact of the paper seems to be negatively related to the proximity of its authors to all other authors in the network. As expected, instead, *author degree* and *author betweenness* co-vary with the citations received by the articles, thereby connecting distant authors and having a large number of co-authors co-occur with higher citations, and they are also positively correlated (0.81 with p<0.001), as it occurs in most networks [39].

We also find that *article degree proportion*, *article betweenness* and *article clustering coefficient* are positively correlated with the citations received (.22 and .30 respectively with p<.001) and between each other (.70 with p<.001). The *article degree proportion*'s value show that there is a link between size of the research community to which the article belongs and its *citation count*, and *article betweenness* correlation show that bridging fragmented strands of literature usually signals an increase in the *citation count*. The positive value between *article clustering coefficient* and *citation count* shows that being embedded in a literature co-varies with citations. Also control variables such as the *number of authors*, the *size of literature* and the *author*'*s experience* are positively and significantly correlated with the citation count.

In the next paragraph we present the results of the regressions computed on three models. In model 1, we replicate a part of a recent study that analyzed the structural effect of the co-authorship network on the *citation count* [2]. In model 2, we add the BC measures, whereas model 3 is generated on a smaller dataset that considers articles published between 2008 and 2010, a period in which the giant component – the largest component of the network in which all nodes can be linked by a path – in co-authorship network connects a relevant portion of the nodes. To control for outliers, we exclude the first three observation for their abnormally large score of *Author closeness* that is due to the number of nodes in the CA (the first three observations take values of 1 and .33, whereas the fourth largest observation of .038 – as shown in figures S1 and S2 in File S1).

All regressions are performed with the negative binomial regression model that is appropriate for count-data models and has no specific assumption on the dependent variable, unlike the Poisson and the zero-inflated Poisson. Poisson assumes that the mean and the variance of the dependent variable should be equal, while in our dataset they differ significantly (µ = 4.952, σ^2^ = 97.8116). Nonetheless the large number of articles in the dataset whose *citation count* takes value 0, we still prefer the negative binomial model to a zero-inflated Poisson, because the latter model assumes that many of the observations that take value zero are drawn from a different distribution in which articles will never be cited. In our case, there is no theoretical reason to assume that non cited articles come from a different distribution. Results are displayed in Table 3.

**Table 3:** Results of regression analysis.

	model 1	model 2	model 3
	Estimate	Estimate	Estimate
Intercept	0.73†	0.64†	0.45
	*0.06*	*0.06*	*0.31*
Author degree	0.02†	0.02†	0.01†
	*0.00*	*0.00*	*0.00*
Author betweenness	−52.88†	−51.98†	−21.60
	*14.61*	*14.59*	*14.86*
Author closenesss	−9.03	−4.63	3161.00†
	*10.74*	*10.93*	*891.30*
Article degree proportion		−0.93	0.36
		*0.81*	*0.97*
Article betweenness		0.82**	0.17
		*0.25*	*0.41*
Article clustering coeff.		0.30*	0.24
		*0.12*	*0.16*
New topic generation	−0.29.	−0.26	
	*0.16*	*0.16*	
New topic combination	0.08	0.09	0.00
	*0.07*	*0.07*	*0.10*
Number of authors	0.08†	0.08†	0.07†
	*0.01*	*0.01*	*0.01*
Size of Literature	3.91E-05**	3.530E-05*	-3.29E-06
	*1.36E-05*	*1.409E-05*	*4.67E-05*
Review	0.72†	0.69†	0.64†
	*0.05*	*0.06*	*0.07*
Author's experience	0.03*	0.02.	0.00
	*0.01*	*0.01*	*0.01*
Bandwagon effect	0.08	0.05	0.02
	*0.10*	*0.10*	*0.13*
Observations	3340	3340	1684
Log-likelihood	−8505.26	−8498.22	−4509.65
McFadden R2	0.04	0.04	0.04

Signif. Codes: ‘†’ p<0.001; ‘**’ p<0.01; ‘*’ p<0.05; ‘.’ p<0.1. Standard errors in italics. McFadden r squared is 1 - (loglikelihood (model)/loglikelihood (intercept))

### The impact of co-authors' network position on the article's citation

*Author degree* has a positive highly significant but very small coefficient (between 0.02 and 0.01) throughout the three models. Such a positive association on citations remains when we add also the network metrics involving the shared references (in model 2), and is robust also in the smaller dataset comprising the articles published between the 2008 and 2010. This result shows that articles written by authors who have established more co-authorship relations (with other authors in the dataset) tend to be cited more. It must be noted that this cannot be a strategy pursued by authors, as the increase in citations generated by each relation is extremely small. Surprisingly, and unlike other studies [2], [7], *author betweenness* is negatively and significantly associated with the citations received by the paper as long as the giant component connects a relevant part of the authors in the dataset (model 1 and 2 versus model 3). The coefficient is large, because the normalized betweenness scores take extremely small values (min 0, max 0.025, with a right skewed distribution in which 75% of the values are below 6 · 10^−7^). The intuition is that bridging between groups of co-authors is negatively associated to *citation count*. It is not clear the reason underpinning such a result: it could be due to the experience of the bridging author and to the mathematical construction of the measure of betweenness. Regarding the experience of the bridging author, research in cognitive science says that a long and vast experience is necessary to successfully put together different pieces of knowledge [40], [41], thereby we could think there may be some interaction between betweenness and authors' experience. Moreover the mathematical representation of the metric of betweenness overemphasizes the size of the population groups that are linked, while neglecting the redundancy of edges, thereby not capturing the number of different knowledge bases. For example, assuming that different groups have different expertise, betweenness does not distinguish if an author creates a bridge between two numerous groups (two knowledge domains), or alternatively more groups (more knowledge domains) but less numerous. Furthermore betweenness is equally sensitive to nodes directly and indirectly connected to the author, thereby it is also dependent to the size of the component. Thereby one author's betweenness score is high if she sits in a large network, despite that only a very limited number of them are directly in contact. For this reasons, we believe that *author betweenness* becomes not significant when we reduce the observation to those in presence of a relevant giant component, see Table 4. Although the score is high, it does not reflect the personal benefit that a person can acquire from sitting in a position more ‘in between’ within the network. Results then suggests us not to be conclusive in our conjectures and instead suggest carrying out further analysis to understand whether this or other measures should be used to capture the idea of creating bridges across groups.

**Table 4:** Number of authors in the Giant Component (GC) with respect to the total authors in the network.

	1998	2000	2002	2004	2006	2008	2010
Authors in the field	594	1003	1429	2222	3461	5398	8590
Authors in the GC	79	94	98	231	723	1565	3589
Percentage in the GC	13.30%	9.37%	6.86%	10.40%	20.89%	28.99%	41.78%

Results of model 1 and 2 suggest that there is no association between *author closeness*, i.e., the proximity of an author to all others in the network, and the *citation count*. However, *author closeness* is sensitive to the structure of the CA. Initially the lack of collaboration between groups does not generate a unified network structure and authors cannot use their network of relations to usefully disseminate their work. Closeness plays a positive role in the presence of a tangible giant component (see Table 4). Model 3 shows the results of the regression on the subset of 1683 articles published between 2008 and 2010 where *author closeness*'s coefficient becomes positive and significant (3161 with p-value  = 0.0004), proving that closeness is positively associated with *citation count*, and this may be due to the fact that direct forms of social interaction – co-authorships – facilitate the diffusion of ideas.

In summary, data support the idea that authors benefit from their accumulated co-authorship relations, and that being embedded (better when in the core rather than in the periphery) in a large network boosts the citation count of the paper. Betweenness centrality instead appears to be a problematic measure to analyze the combination of knowledge occurring in the single paper.

### The impact of the bibliographic coupling network

**Figure 5:**
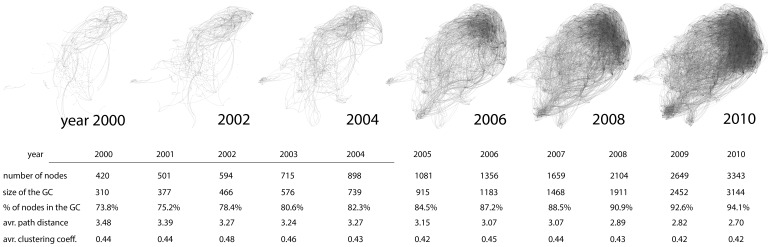
The evolution of the Vulnerability literature with the Bibliographic Coupling network. Nodes are represented by articles in the dataset and the edges link articles which share one or more references. In this network, we show only articles with at least 10 citations and that are connected to the giant component (1164 nodes in 2010 that are 72,48% of amount of articles with more than 10 citations).

Model 2 shows that *article betweenness* is positively associated to the *citation count* (0.82, p-value <0.01) and highlights the scientific value of those works that find connections among others that are already present in the literature. It draws the attention to the fact that looking for relationships among theoretical arguments and finding connections with remotely connected or yet disconnected knowledge domains and theories is rewarded in terms of citations, even in the short term of two years. When we restrict the analysis to the articles written from 2008 to 2010, and the giant component comprises over 90% of nodes, it seems less obvious the advantage to find new ties with the existing literature, moreover there are fewer groups of nodes to incorporate in the main component. Thus the effect of *article betweenness* on citations becomes not significant when the giant component in the BC absorbs most nodes, as shown both in the regression of model 3 and in Figure 5.

Similarly, *article clustering coefficient* shows that being embedded in a literature benefit the *citation count* of the article (0.30 with p-value <0.05), but the effect disappears when computed for the articles written after 2008. One possible reason is that an increasing homogeneity of articles' references could be forced by exogenous pressures thus reducing the positive effect of the embeddedness. This hypothesis could be reinforced by the call for integration of the concept of vulnerability among various streams of literatures made by international bodies of research such as UNISDR and IPCC.

*Article degree proportion*, a proxy of the size of the research community, does not significantly impact the *citation count*. Thus we can conclude that, at least in this dataset, sharing references with a large number of other articles in the extant literature is not a practice that increases the number of citations received, suggesting that recognizing arguments already well spread in the literature by acknowledging very popular prior research, as well as the size of the research community, have no apparent relation with the scientific impact. Therefore, these practices that are typical of positioning a paper within a literature sort no significant effect at the citation side.

Curiously, among the control variables, *topic generation* is negatively associated to the number of citations, although in model 2 the p-value is slightly larger than 10%, while there is no effect for the *new topic combination*. The result of a new topic is not due to a different distribution of citations over the years (see SI), but it may be due to the fact that when vulnerability was brought into different topics, the formalization of the construct was still fuzzy, thus articles did not benefit from this inclusion. Only after the year 2002, there have been the most prominent theoretical advancements that established the concept and provided useful frameworks[42], [43]. However, topic generating articles are concentrated before the year 2000.

The *number of authors*, the *type of article* (review or non-review) and the *size of literature* are all positively and significantly associated with citations, and especially review articles have a large positive effect on citations. When we control for the position of the article in the literature, the *author*'*s experience* and the *size of literature* lose significance, showing that, at least in this setting, the number of articles published in the field is not a good predictor for the success of the next article, nor is the size of the citing community. In our setting, there is no *bandwagon effect* or, if there is, it is not extended to all co-authors of the most cited papers. This property is more likely to reside in a handful of them (perhaps the first and last one) who can on the one hand produce outstanding articles, and on the other benefit from peer-recognition.

## Discussion and Conclusion

In this work, we analyzed the effects on citations of the social and knowledge networks on which scientific articles are grounded. This work extends the knowledge accumulated on the effects of the co-authorship network on the scientific impact [2], [3], [7], [15], and tries to ground it better in the theory of knowledge combination upon which it is based [12], [14], [41], [44], by bringing in a second type of network that analyzes the knowledge sources. In particular, we felt compelled to answer two research questions: (1) how does the co-authorship network structure influence the scientific impact of an article in terms of citations? And (2) how do the article's knowledge sources influence its scientific impact? To answer these questions, we retrieve the articles published in the scientific literature on vulnerability of social and natural environment due to climate change and natural hazards. Then we construct the dynamics of the co-authorship and bibliographic coupling networks, based on co-authorship relations among authors and shared references among articles respectively.

With no surprise, we find that the structure of scientific collaborations matters. The cumulative number of co-authors has a positive – yet slight – impact on the citations of the article, while a larger and positive effect is given by the proximity of authors to all others in the field. However, the effect due to the proximity manifests itself only when it is possible to trace a collaboration link among a relevant share of authors in the field. This is consistent with the idea that knowledge diffusion is aided by personal relationships which could be costlessly and effortlessly tapped. Surprisingly, and in contrast to other studies [2], [7], we found that bridging among groups of authors is penalized in terms of citations. Such result is counterintuitive, because the intuition would suggest that authors who create a bridge among different groups, also bridge among their knowledge bases, thus their ideas could benefit from the eventual distance among knowledge domains. We believe that the intuition, and the theory, still hold, but we propose two reasons that could explain such a result. A first reason that could moderate the negative impact of bridging could be due to experience of the author who establishes ties with other groups, as studies in cognitive science claim [14], [40], [41]. The second reason is regards the measure of betweenness centrality, adopted in this and similar studies: betweenness centrality on the one hand overemphasizes the effect of the indirect connections and thus depends on the size of the component and on the other neglects the redundancy of edges among nodes. Therefore it does not reflect the number of diverse knowledge domains a single author can benefit from. Other measures could be explored in future research and a promising perspective could be given by an adaptation of the clustering coefficient computed over sets of nodes (the papers' co-authors), instead on individual ones. In other words, when two authors decide to work together, their respective networks get together, but they are still separated by the edge that links the two coauthors. Therefore, knowledge diversity must be computed counting the common ties between every possible dyad among all prior co-authors of the authors.

With regard to knowledge sources, we found that articles receive more citations when the authors are aware of what happens in different strands of literature, demonstrated by the references included in their article, and are able to make a synthesis between the preexisting and disconnected ideas. Thereby we claim that articles that find ways to tie together fragmented pieces of literature obtain more citations. Also we found that articles receive more citations when they are positioned in a literature that builds on a common base of articles.

Neither a specific benefit, nor a disadvantage comes from the size of the research community. We identified the size of the research community by looking at the practice adopted by authors who signal their belonging to a community by citing what ‘similar’ papers cite, thus boosting the degree centrality in the bibliographic coupling network.

Besides the use of the bibliographic coupling network, the adoption of topic modeling is a second innovative methodological component of this work. We used this tool to sort a large set of literature and control for innovative papers in terms of their content. We want to stress the utility of the tool in describing the findings – some unexpected – related to the topic variables. We saw that topic generating articles are concentrated in the early years (1989–2002) when the literature is still fragmented: the co-authorship network and bibliographic coupling network are made of many components. These are signs that the body of literature on vulnerability does neither cohesively grow upon seminal contributions nor as a unified body of literature whereby authors speak to each other. Instead, articles belong to different and separate domains (probably pertinent to the topics as pre-existing and different strands of literature) that introduce the concept of vulnerability in different years. We may conjecture that they do not benefit from importing the concept of vulnerability in their literature, perhaps because the concept is still fuzzy, differently defined in different domains, and seminal contributions had yet to come. As regard topic combination, the vast nature of the dataset allowed the identification of relatively distant topics such as drought, river basins and arctic, whose combination in the same article may signal a generic approach and wide scope rather than a provision of specific and innovative contributions. We believe that peer recognition comes from deep analyses and novel results: features that still elude our algorithms.

Indeed, we recognize that this work comes with limitations, which drive also ideas for future research. As already mentioned, the negative impact of the bridging author is yet unclear and future research should be carried out to discover why there seems to be no advantage to broker. We propose that future research may adopt team-adjusted measures of network constraints [45], or clustering coefficient computed on the redundancies of the ties of sets of nodes.

We are aware that a second limitation is due to the large number of journals and edited books (1079 sources) that impeded us to control for possible journal effects on citations. We think that having a control for journals would absorb some of the variability in the data. However, such a limitation does not affect the validity of results that focused on the effect of co-authorship and bibliographic coupling network structures on the early citation received.

Another limitation comes with the simplification. We attributed the value given by the co-author with highest centrality scores to each article, and this prevented us from analyzing the information given by the heterogeneity of the set of co-authors. However, in this work, we think we have accomplished a first step to reconcile the often concealed knowledge aspect of generation of scientific knowledge with the more studied social one.

## Supporting Information

File S1 Contains the files: Figure S1 – Scatterplot of the Bibliographic Coupling network data. Figure S2 – Scatterplot of the Co-authorship network data.
(DOCX)
