# Supplementary material for: Co-Authorship and Bibliographic Coupling Network Effects on Citations
Source: PLoS One. 2014 Jun 9;9(6):e99502. doi: 10.1371/journal.pone.0099502 (PMC4049820; doi:10.1371/journal.pone.0099502)
Supplement: File S1 — Contains the files: Figure S1 – Scatterplot of the Bibliographic Coupling network data. Figure S2 – Scatterplot of the Co-authorship network data. (DOCX) [file pone.0099502.s001.docx]

SUPPORTING INFORMATION

Discovering topics from articles

Scanning and separating large collections of documents, the corpus, according to their underlying themes can be grueling and extremely time consuming. However recent developments in computer science applied to Natural Language Processing ([Blei, 2012](#_ENREF_1); [Blei, Ng, & Jordan, 2003](#_ENREF_4); [Chang & Blei, 2010](#_ENREF_5)) have enabled the discovery of the hidden thematic structure of large sets of documents through the analysis of the observables: words and their allocation in the corpus.

The intuition is that every document reflects one or multiple topics. For example an article on vulnerability may have some parts of mathematics, some others of evolutionary biology, and some of economics. As a consequence a document can be seen as a distribution over topics, whereas a topic is a probability distribution over the whole set of words in the vocabulary (i.e., the set of words adopted in the whole collection of documents in the dataset). In Natural Language Processing, the idea of describing the contribution of different topics to a document is commonly modeled through two the iterative interaction of joint probability distributions: the random extraction of the word *w_i_* from a topic, $P\left( w_{i} | z_{i}=j \right)$, that is multiplied by the probability of picking that from the topic *j,* $P(z_{i}=j)$ ([Blei & Lafferty, 2006](#_ENREF_2); [Blei & Lafferty, 2007](#_ENREF_3); [Blei et al., 2003](#_ENREF_4); [Griffiths & Steyvers, 2004](#_ENREF_6)). And if there are *T* topics, the probability of the *i-*th word is given by $P\left( w_{i} \right)=\sum_{j=1}^{T} P\left( w_{i} | z_{i}=j \right)P(z_{i}=j)$. The intuition is that $P\left( w | z \right)$ gives the idea of the importance of words in topic, whereas $P(z)$ is the probability to find a topic in a document.

The origin of the generative such algorithm, the Latent Dirichlet Allocation (LDA), underpinning topic modeling is to classify individuals in groups according to their genetic expressions. In the analogy of topic modeling adapted to texts, individuals are like *documents*. They contain a combination of expressions or alleles, that are observable, like words. While the group is the latent variable that must be inferred, therefore it is the analog of the *topic*. Groups are distributions over the alleles ([Pritchard, Stephens, & Donnelly, 2000](#_ENREF_7)).

The LDA algorithm requires to specify the number of topics, then the algorithm tries to maximizing the joint probability $P\left( \boldsymbol{w} | \phi,\theta\right)$ that describes the equation $P\left( w_{i} \right)=\sum_{j=1}^{T} P\left( w_{i} | z_{i}=j \right)P(z_{i}=j)$ computed for all words in a document as well as for all documents. $\phi$ is a set *T* multinomial distributions of each word in the document and describes the probability of each word to be generated by the topic distribution. Whereas $\theta$ is a set of *D* multinomial distributions over the *T* topics (for a simple and more exhaustive explanation see Blei, 2012).

The algorithm requires the specification of two parameters α, topic smoothing, and β, term smoothing, that we held = .01 (like in Kaplan and Vakili 2012) to guarantee a fair granularity in the results and a clear attribution of topics per document. However, the topic modelling algorithm assumes that a document is a “bag of words”, whereby word order is irrelevant. An unrealistic assumption for language generation, but sufficient for revealing the hidden content of topics. Moreover, it assumes that the order of documents in the list does not matter and topics do not change over time, and in our case this is irrelevant because of the documents are written in a relatively short period of time. When they span over many years or centuries, it is possible to define topics as series of distributions over words and see how they change over time ([Blei & Lafferty, 2006](#_ENREF_2)).

Among the applications, topic modeling has already been adopted to draw relations among scientific articles based on their thematic similarity ([Chang & Blei, 2010](#_ENREF_5)). In this research, we use topic modeling to categorize the whole corpus of scientific articles and sift out the non-relevant ones that were still present despite the keyword selection. This operation prevents us from comparing articles of different disciplines.

Topic Classification

In Supplementary Table 1, we show the topics names given by the three experts and their discussion on whether to keep the articles within the topic or not. Parentheses, such in the case of Topic 14 [Seismic risk] and Topic 20 – [Public Governance and Crises] are determined by the difficulty in labeling the topic. While the two question marks in the Keep column have required a qualitative check on the papers to decide whether to keep the articles with predominant text on that topic or to exclude them from the dataset. They have been kept because in the community of Disaster Risk Reduction, the term Vulnerability is often associated to studies on seismic activities. The decision was similarly taken also for topic 20, because of the crucial role of Public institutions in risk mitigation.

**Supplementary Table ST1 – Topic list**

| Code | Title | # of words | Keep |
| --- | --- | --- | --- |
| **Topic 00** | **Decision making & Information management** | **8379** |  |
| **Topic 01** | **Global processes & issues** | **10047** |  |
| **Topic 02** | **Vegetation & drought** | **7524** |  |
| **Topic 03** | **Nature conservation & biodiversity** | **5739** |  |
| **Topic 04** | **Natural hazards & DRR** | **8024** |  |
| **Topic 05** | **Supply chains & business** | **2946** |  |
| **Topic 06** | **Ecology & animals** | **4311** |  |
| **Topic 07** | **Landslides** | **5691** |  |
| **Topic 08** | **Weather extremes** | **5836** |  |
| **Topic 09** | **Spatial (& temporal) scales** | **7474** |  |
| **Topic 10** | **[Tourism]** | **4797** | **N** |
| **Topic 11** | **Weather extremes 2** | **4661** |  |
| **Topic 12** | **Human dimension** | **7419** |  |
| **Topic 13** | **Time** | **9430** |  |
| **Topic 14** | **[Seismic risk]** | **9512** | ? |
| **Topic 15** | **Spatial analysis** | **8261** |  |
| **Topic 16** | **Regional analysis** | **6496** |  |
| **Topic 17** | **Health** | **7219** |  |
| **Topic 18** | **Resilence and (SE)systems** | **9093** |  |
| **Topic 19** | **[Volcanos (islands)]** | **5415** | N |
| **Topic 20** | **[Public gov and crises]** | **6238** | **?** |
| **Topic 21** | **Programmes & projects** | **7642** |  |
| **Topic 22** | **Soil science** | **6016** |  |
| **Topic 23** | **Cities** | **4963** |  |
| **Topic 24** | **[Medicine: infective diseases]** | **3687** | N |
| **Topic 25** | **Ecology (birds)** | **5692** |  |
| **Topic 26** | **Ecosystems** | **6152** |  |
| **Topic 27** | **Economics** | **6489** |  |
| **Topic 28** | **History (drought)** | **8177** |  |
| **Topic 29** | **[Medicine: stresses]** | **5613** | **N** |
| **Topic 30** | **Coasts** | **6154** |  |
| **Topic 31** | **Sustainability & resources** | **5659** |  |
| **Topic 32** | **Policy strategies & measures** | **14317** |  |
| **Topic 33** | **Capacities & adaptation** | **8967** |  |
| **Topic 34** | **Coasts & seas (SLR)** | **7506** |  |
| **Topic 35** | **Temperatures** | **6462** |  |
| **Topic 36** | **Systems (model)** | **6698** |  |
| **Topic 37** | **Flood** | **5092** |  |
| **Topic 38** | **Heat waves** | **5887** |  |
| **Topic 39** | **Carbon in soils** | **5156** |  |
| **Topic 40** | **Forests** | **6223** |  |
| **Topic 41** | **Extremes & DRR (hurricanes)** | **4739** |  |
| **Topic 42** | **Africa & LDCs** | **4122** |  |
| **Topic 43** | **Socio-political issues** | **8589** |  |
| **Topic 44** | **[Medicine]** | **5613** | N |
| **Topic 45** | **Tsunami** | **4349** |  |
| **Topic 46** | **Ice cap conditions & Arctic** | **4082** |  |
| **Topic 47** | **Scenarios & projections** | **11751** |  |
| **Topic 48** | **Local communities & stakeholders** | **8238** |  |
| **Topic 49** | **Coasts and fisheries** | **5881** |  |
| **Topic 50** | **Groundwater** | **8737** |  |
| **Topic 51** | **[Infrastructures]** | **5330** |  |
| **Topic 52** | **Methods & frameworks** | **13130** | **N** |
| **Topic 53** | **Generic words** | **10393** |  |
| **Topic 54** | **Indicators & indices** | **10400** |  |
| **Topic 55** | **Agriculture** | **7130** |  |
| **Topic 56** | **Watersheds, rivers and basins** | **7165** |  |
| **Topic 57** | **Mediterranean & Europe** | **5896** |  |
| **Topic 58** | **Generic security** | **4985** |  |
| **Topic 59** | **Generic seasons** | **4269** |  |
| **Topic 60** | **Losses & damages** | **6640** |  |
| **Topic 61** | **[Industrial safety]** | **5124** | **N** |
| **Topic 62** | **Demography** | **6249** |  |
| **Topic 63** | **Energy, emissions & mitigation** | **7101** |  |
| **Topic 64** | **Ecology (habitat)** | **8970** |  |
| **Topic 65** | **Lakes & freshwater** | **4312** |  |
| **Topic 66** | **Food (security)** | **7742** |  |
| **Topic 67** | **Fires** | **3749** |  |
| **Topic 68** | **[Medicine]** | **9266** | **N** |
| **Topic 69** | **[Medicine]** | **5888** | N |
| **Topic 70** | **USA** | **4481** |  |
| **Topic 71** | **Agriculture (crops)** | **6767** |  |
| **Topic 72** | **Models & uncertainty** | **10345** |  |
| **Topic 73** | **Climate variability** | **7019** |  |
| **Topic 74** | **Reserach & science** | **10915** |  |

In Supplementary Table ST2, we show a sample of topics and the data with which experts decided to attribute titles to the topic and their relevance to the field

**Supplementary Table ST2 – 20 most frequent words for a sample of topics**

| **Topic 00** | **Topic 04** | **Topic 46** | **Topic 56** | **Topic 65** |
| --- | --- | --- | --- | --- |
| *Decision making & information management* | *Natural hazards & Disaster Risk Reduction* | *Ice cap condition & Arctic* | *Watersheds, rivers and river basins* | *Lakes and freshwater* |
| decision | disaster | ice | river | lake |
| information | hazards | arctic | basin | lakes |
| making | natural | sea | resources | stream |
| support | disasters | inuit | hydrological | aquatic |
| system | hazard | conditions | watershed | fish |
| tool | reduction | shelf | runoff | streams |
| makers | mitigation | alaska | basins | river |
| gis | preparedness | reindeer | flow | during |
| integrated | prevention | traditional | catchment | salmon |
| tools | emergency | local | rivers | structure |
| planning | human | nunavut | hydrologic | regime |
| decisions | government | subsistence | flows | salinity |
| application | causes | community | scarcity | quality |
| provide | prone | processes | watersheds | summer |
| useful | measures | canada | streamflow | freshwater |
| knowledge | important | warming | hydrology | conditions |
| multi | risks | changing | availability | food |
| available | through | peninsula | discharge | response |
| stakeholders | people | ocean | storage | flow |
| managers | reducing | hunting | reservoir | increase |

Statistical Analysis

Scatterplots of citations and network metrics display the presence of three outliers that were excluded from the statistical analysis. These outliers are clearly visible in the closeness centrality boxes at the bottom of Figure S2. The values of these three elements are abnormal with respect to the distribution of the closeness scores of the others (the first three observations take values of 1 and .33, whereas the fourth largest observation of .038), due to the extremely low population of authors in the early stage of the literature.

Figure S1 - Scatterplot of the Bibliographic Coupling network data


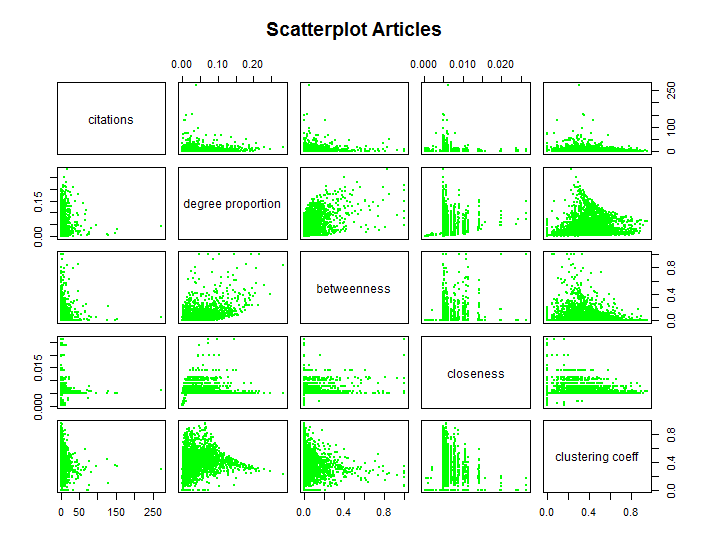


Figure S2 – Scatterplot of co-authorship network data


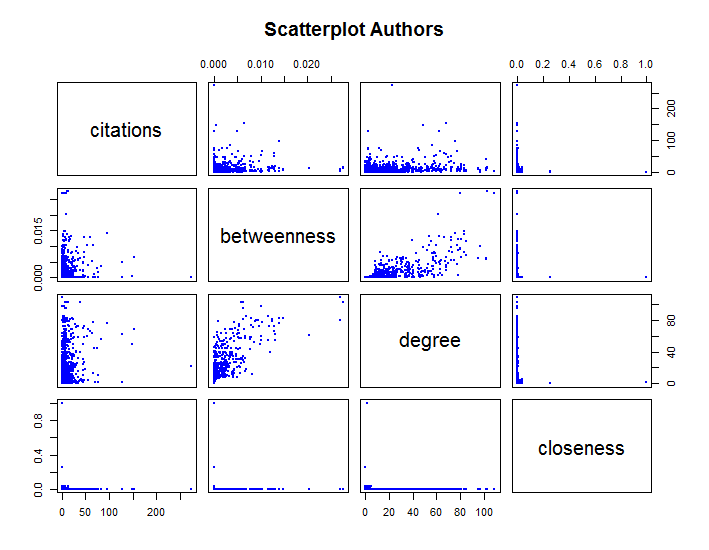


References

Blei, D. M. 2012. Probabilistic Topic Models. ***Communications of the Acm***, 55(4): 77-84.

Blei, D. M., & Lafferty, J. D. 2006. ***Dynamic topic models***. Paper presented at the Proceedings of the 23rd international conference on Machine learning.

Blei, D. M., & Lafferty, J. D. 2007. A correlated topic model of science. ***Annals of Applied Statistics***, 1(1): 17-35.

Blei, D. M., Ng, A. Y., & Jordan, M. I. 2003. Latent Dirichlet allocation. ***Journal of Machine Learning Research***, 3(4-5): 993-1022.

Chang, J., & Blei, D. M. 2010. Hierarchical relational models for document networks. ***The Annals of Applied Statistics***, 4(1): 124-150.

Griffiths, T. L., & Steyvers, M. 2004. Finding scientific topics. ***Proceedings of the National Academy of Sciences of the United States of America***, 101(Suppl 1): 5228-5235.

Pritchard, J. K., Stephens, M., & Donnelly, P. 2000. Inference of population structure using multilocus genotype data. ***Genetics***, 155(2): 945-959.
